# Bioenergetic Evaluation of Muscle Fatigue in Murine Tongue

**DOI:** 10.1007/s00455-022-10537-y

**Published:** 2022-11-19

**Authors:** Tiffany J. Glass, Linda M. Rowe, Jared Cullen, Nadine P. Connor

**Affiliations:** 1 Department of Surgery, Division of Otolaryngology, University of Wisconsin Madison, 483 Medical Sciences Center (MSC), 1300 University Ave, Madison, WI 53706, USA; 2 Department of Communication Sciences and Disorders, University of Wisconsin Madison, Madison, WI, USA

**Keywords:** Fatigue, Tongue, Murine, Ageing, Exercise, Dysphagia

## Abstract

Muscle fatigue is the diminution of force required for a particular action over time. Fatigue may be particularly pronounced in aging muscles, including those used for swallowing actions. Because risk for swallowing impairment (dysphagia) increases with aging, the contribution of muscle fatigue to age-related dysphagia is an emerging area of interest. The use of animal models, such as mice and rats (murine models) allows experimental paradigms for studying the relationship between muscle fatigue and swallowing function with a high degree of biological precision that is not possible in human studies. The goal of this article is to review basic experimental approaches to the study of murine tongue muscle fatigue related to dysphagia. Traditionally, murine muscle fatigue has been studied in limb muscles through direct muscle stimulation and behavioral exercise paradigms. As such, physiological and bioenergetic markers of muscle fatigue that have been validated in limb muscles may be applicable in studies of cranial muscle fatigue with appropriate modifications to account for differences in muscle architecture, innervation ratio, and skeletal support. Murine exercise paradigms may be used to elicit acute fatigue in tongue muscles, thereby enabling study of putative muscular adaptations. Using these approaches, hypotheses can be developed and tested in mice and rats to allow for future focused studies in human subjects geared toward developing and optimizing treatments for age-related dysphagia.

## Introduction

Across the globe, people are living longer and populations are aging. It is estimated that by the year 2030, 1 in every 8 people globally will be aged 65 or older [1]. This demographic shift is associated with challenges in ensuring the health and well-being of older adults. Dysphagia is disproportionately higher in older adults [2, 3], incurring a need for expansion of treatment options for age-related dysphagia. A necessary step in ensuring the development of effective treatments is a clear understanding of the characteristics and mechanisms of aging cranial muscles that contribute to swallowing actions, and how aging may impact functional task performance, such as maintenance of endurance while eating a meal.

Fatigue is a common complaint in older adults for activities of daily living, and at mealtimes [4, 5]. Asymptomatic changes to swallow function caused by age-related changes in structure and physiology of the upper aerodigestive tract—known as presbyphagia—can predispose older individuals to develop dysphagia [6]. Muscle fatigue, which may be defined as a failure of the muscle to maintain a required force, may be a contributing factor to age-related dysphagia [7] when found in key muscles that support swallowing actions, such as muscles of the tongue. Although tongue exercises are often used in dysphagia management [8–10], relatively little is known about how tongue exercise impacts muscle fatigue. This knowledge gap is attributable to inherent challenges of clinical studies, including considerable variability in human participants, and limitations to direct analysis of muscle biology and physiology of human cranial muscles.

Animal models provide a means to study tongue muscle biology with a high degree of experimental precision that is not possible in humans. Mice and rat (murine) models offer several strategic advantages for this area of research, and a variety of experimental options for the study of the biology of tongue muscle fatigue. This review presents the relationship between fatigue and altered swallow function with age and provides an overview of experimental approaches currently available for the study of tongue muscle fatigue in murine models. It is anticipated that future efforts for treatment development specifically targeting age-related dysphagia may in part draw upon this emerging interdisciplinary area of research to allow development of hypotheses that can be tested in humans.

## Muscle Fatigue and Presbyphagia

Although fatigue is a common complaint in older adults, it can be difficult to define due to many variables that affect where a breakdown in function may occur. In the absence of a likely underlying etiology, an older adult experiencing muscle fatigue may be evaluated for generalized weakness, frailty, sarcopenia, or dynapenia [11–13]. Common complaints colloquially associated with fatigue in older adults include tiredness or weakness [14], and difficulty performing activities of daily living[15, 16], which elevates the individual’s risk of negative health outcomes, reduced quality of life, and loss of independence [17]. As previously reviewed, aging results in several processes that impact muscle fatigue[18, 19], including reduced numbers of motor neurons [20] that in turn each innervate a greater number of myofibers [21], reductions in myofiber size [22], changes to the neuromuscular junction [23], and changes in muscle fiber type [24]. Collectively, these alterations may lead to reduced strength of muscle contraction, decreased stability in performance of repeated muscle contraction, and a decrease in functional reserve [18, 25] that can significantly impact an older individual’s ability to safely perform daily activities.

Age-related changes to the structure and function of the upper aerodigestive tract can result in changes to deglutition (presbyphagia) that predispose an individual to impaired safety or efficiency of deglutition [26]. Clinical studies have identified age-related reduction in efficiency of peak pressure generation [27], delayed swallow onset [28], longer duration of upper esophageal sphincter (UES) opening [28], reduced lingual pressure reserves[29], and alterations to timing of respiratory-swallow events [30–32]. Translational studies allowing for biological and physiological analysis of swallow musculature have identified a transition toward slower-twitch muscle fibers in the extrinsic tongue with age [33, 34], slower contraction time to reach maximum peak force[35], and degenerative changes to afferent and efferent nerve fibers [36]. These age-related changes to deglutition can occur in presbyphagia without explicit deleterious effects on overall swallow function [37]. However, these reductions in biological, physiological, and functional reserves increase an older individual’s vulnerability to dysphagia in the presence of other factors or comorbidities such as stroke or neurodegenerative disease [6, 38]. Dysphagia in these populations can lead to significant risk of negative health outcomes including aspiration, pneumonia[39], malnutrition, dehydration, and reduced quality of life [40–42].

Although the tongue has been considered indefatigable for swallow tasks (given that maximum force capacity of the tongue exceeds the minimum amount of force required for these tasks [43]), it is known that performance of oral motor tasks can be impacted by fatigue [44]. Studies have shown that performance of oral motor tasks can be impacted when muscles are brought to fatigue prior to initiating a functional oral motor task, indicating that fatigued tongue muscles demonstrate impaired performance on motor speech[45] and swallowing tasks [46] when muscle reserves are depleted. Further, in a study evaluating the impact of eating on tongue strength, measures of tongue strength and endurance in older adults were reduced after eating a meal, suggesting that consumption of a typical meal may be construed as an endurance activity [7]. In a subsequent study, when tongue muscle fatigue was elicited through the use of a repetitive tongue pressure task, both young and older adults demonstrated a direct impact of fatigue on measures related to deglutition after consuming a meal [46]. Specifically, participants repeatedly exerted target tongue pressures using an Iowa Oral Performance Instrument^®^ (IOPI; IOPI Medical LLC) device until experiencing an inability to sustain a target force. It was found that participants who consumed a meal following elicited tongue fatigue had significant post-meal decrements in posterior tongue pressures, which is compatible with the possibility that tongue fatigue may influence swallowing safety during meals. Further, older adults reached tongue fatigue more quickly than young adults, and had significant differences in multiple post-meal measures related to swallow function than younger adults; recapitulating prior findings of age-related differences [46]. Collectively, these studies illustrate the likelihood that aging is associated with functional changes that can compromise mealtime safety, and fatigue of tongue muscles may be integral to many of these changes.

## Tongue Muscle Fatigue

Tongue muscles have fundamentally different structure and function than limb muscles [47]. The intrinsic tongue is a muscular hydrostat with supportive attachments of inter-digitating muscle rather than the tendonous attachments to bone or cartilage as found in muscles of the limbs [48]. The size and type of muscle fibers vary significantly by region within the tongue[49]. These properties allow the tongue to engage in rapid changes to its shape and position for executing highly complex, specialized functions.

The tongue has a dual role in two critical functions that are essential for survival: swallowing and respiration. Its mechanical functions in bolus propulsion and airway patency are highly conserved among mammalian species [50]. Although respiratory muscle reserves are reduced with aging, making them less robust to challenges, respiratory muscles maintain adequate ventilation throughout the lifespan under typical conditions [51]. As a dual muscle of respiration, it is possible that the tongue may demonstrate a fatigue resistance profile more consistent with other respiratory muscles than with the limb.

Studies have evaluated fatigue in both limb and tongue where participants exerted repeated presses of 50% maximum force before and after acutely fatiguing the hand and tongue with maximal contractions. Following acute fatigue, the tongue demonstrated a significantly higher rate of force decay than prior to the fatigue induction and this finding was replicated across studies [52, 53]. In contrast, hand pressure force decay rates following fatigue were not well replicated across studies, which was attributed to individual variability[52, 53]. It was unclear whether central or peripheral mechanisms were primarily responsible for the tongue fatigue in these studies and it was suggested that future studies of direct electrical stimulation of nerve or muscle could assist in identifying the “locus of fatigue” [53].

A study of direct stimulation of tongue and limb muscles in rats found that the tongue muscles were notably resistant to fatigue, in comparison to limb muscles [54]. In a study that compared muscle contractile properties in rat tongue and hindlimb muscles, aging was associated with reduced tetanic forces in the hindlimb muscle, but with typical contraction times [55]. Conversely, in the same animals, tongue muscles retained normal tetanic forces with increased age, but had longer contraction times [55]. Due to the importance of timing of tongue movements to deglutition, these findings underscore the relevance of physiological and biological approaches to the study of age-related dysphagia. It is evident that changes in muscle contractile properties may be differentially expressed in limb versus tongue muscle, indicating a need to specifically assess mechanisms underlying muscle fatigue in the tongue and not rely solely on previous findings from limb muscle studies.

## Exercise Impacts Muscle Fatigability

Exercise can ameliorate decrements in muscle function associated with aging [56, 57]. Exercises that target the tongue directly, such as those that employ a device like the IOPI, and those that activate tongue muscles through increased respiratory drive necessary in aerobic exercise, have potential to impact muscular, functional, and task reserves. The goal of exercise is often to expand these reserves – that is, to increase capacity by widening the gap between muscle force capacity and minimum force *required* for successful task completion, or to increase endurance, which can be described in terms of the amount of time that a required or expected force can be sustained.

Although sustained effort and exercise can induce transitory muscle fatigue from which there is subsequent recovery [45, 53], maintaining an exercise training regimen over time can ultimately promote fatigue resistance in the long term [58–60]. Exercise-induced fatigue resistance has been traditionally studied in limb muscles, but has also been identified within respiratory muscles [61]. The tongue has a significant role in respiratory function [62], and is known to have an adaptive biological response to exercise regimen[63, 64].

Tongue exercise may influence tongue muscle fatigue-ability through an exercise-induced alteration of muscle fiber types from fatigable to less fatigable. A recent study used myofiber staining and direct muscle stimulation to evaluate fatigability of the genioglossus and intrinsic tongue muscles in rats [65]. The genioglossus muscle was comprised of a higher proportion of fatigue-resistant myofibers and was more fatigue-resistant upon muscle stimulation than the intrinsic muscles [65]. In addition, tongue exercise in rats has been associated with muscle fiber type transformations in the genioglossus and intrinsic tongue muscles. In the genioglossus, some studies have reported exercise-induced shifts toward more fatigue-resistant fiber types [66], while others reported no significant differences [34]. In the anterior intrinsic tongue muscles, a shift toward more fatigable fiber types was found following exercise, but more fatigue-resistant fibers were observed in the posterior intrinsic tongue muscles following exercise [64]. The considerable anatomical complexity of the tongue muscle system, as well as the tremendous diversity of exercise regimen parameters and methodological options may contribute to differences in outcomes across studies. Therefore, while it is plausible that tongue exercise may reduce tongue muscle fatigability, this is an area of on-going research motivated by a keen awareness of the potential translational benefits to be gained from an improved understanding of how fatigue occurs in the tongue muscles, and how it can be impacted by exercise. Current knowledge of the impacts of exercise dosing parameters (e.g. frequency, intensity, duration) used in clinical practice is informed by literature pertaining to limb muscle exercise [10], and as such may not be optimized to specifically address tongue muscle fatigue. Controlled studies are required for the development of a dose–response curve specific to tongue exercise, in order to optimize therapeutic efficiency in clinical practice.

## Exercise Paradigms for the Study of Murine Tongue Muscle Fatigue

The study of fatigue mechanisms in human cranial muscle can be difficult to perform due to challenges of controlling variability within a multitude of factors across human beings, and tissue sampling limitations [67, 68]. Murine models of exercise provide two avenues for the study of cranial muscle fatigue. First, intense exertion or exercise can induce acute, or transitory fatigue, which can then be studied by evaluating muscles immediately after exercise. This provides a controlled means to study cranial muscles when they are in a state of acute fatigue. Second, murine exercise paradigms can be used to study exercise as a therapeutic avenue, to evaluate whether particular exercise regimens can ultimately mitigate or prevent cranial muscle fatiguability. Methods for experimental murine exercise paradigms allow the study of exercise-induced lingual fatigue as well as the study of how exercise impacts fatiguability in murine lingual muscles. These exercise paradigms include chronic neuromuscular electrical stimulation, behavioral tongue exercise, and more systemic behavioral aerobic exercises such as treadmill running.

### Chronic Neuromuscular Electrical Stimulation (NMES)

The hypoglossal (XIIth) nerve provides motor innervation to the tongue muscles. Stimulation of the hypoglossal nerve elicits tongue muscle contraction. The experimental chronic NMES paradigm involves surgically implanted bilateral hypoglossal nerve cuffs to permit hypoglossal nerve stimulation in awake rats for controlled sessions occurring intermittently across weeks. Because the hypoglossal nerve is a motor nerve, this NMES in awake rats elicits tongue muscle contraction without eliciting pain or discernible discomfort. This approach permits a model of tongue exercise where the frequency and schedule of the tongue exercise in awake animals can be experimentally controlled and is less subject to the variability intrinsic to volitional, behavioral tongue exercise paradigms.

One option for direct assessments of muscle fatigue is the evaluation of tongue muscle contractile properties in deeply anesthetized rats through direct electrical stimulation of the hypoglossal nerve [54]. After the rat is anesthetized, the tongue is extended, and the tip of the tongue is attached to a suture that is secured to a strain gauge transducer. Fatiguability of the tongue muscle system can then be evaluated and expressed in terms of the reduction in force present after the hypoglossal nerve has been stimulated for a pre-defined period relative to initial force levels [35, 69]. By pairing an approach of chronic NMES exercise intervention with a final assessment of tongue muscle contractile properties, studies of animal models have indicated that chronic NMES can result in reductions in tongue muscle fatigue [33, 70], thereby suggesting a therapeutic avenue to promote resistance to fatigue in tongue muscles.

### Behavioral Tongue Exercise

Non-invasive avenues for eliciting tongue muscle fatigue and modeling tongue muscle exercise in animals include behavioral tongue exercises. As previously reviewed [71], rats can be trained to push the tongue against a disk with a known resistance. Rats can also be trained to engage in a tongue exercise training regimen to gradually increase volitional tongue press forces as resistance at the disk is increased. This paradigm also provides a means to estimate the maximum force that a rat exerts. Progressive resistance tongue exercise in murine models can mimic basic characteristics of tongue exercise paradigms currently in clinical use. In conjunction with muscle contractile property evaluations or biochemical analyses of isolated tongue muscles, tongue exercise paradigms can evaluate how behavioral tongue exercise may impact lingual fatiguability. Prior work using this paradigm in a rat model of aging has reported reduced tongue muscle fatigue following tongue exercise in old rats [66], and the relationship between volitional tongue exercise and biological measures related to tongue muscle fatigue [34]. In this paradigm, tongue exercises occur for a finite period of time each day and may be construed as low-repetition/high resistance tongue exercise regimen. There are other approaches to behavioral tongue exercise in which customized equipment allows automated high-repetition/low resistance tongue resistance training in the rats’ home enclosure, with lower force demands[72]. In addition to these behavioral paradigms that specifically engage the tongue muscles, more systemic exercises can impact tongue muscles in addition to other muscles and may also be used in understanding the relationships between exercise and tongue muscle fatigue. Aerobic exercises, including treadmill running, are an example of an exercise paradigm that may impact tongue muscle biology while also impacting other muscle systems.

### Treadmill Running Impacts Tongue Muscles

**Fig. 1:**
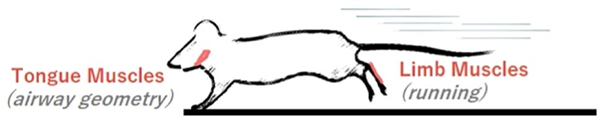
Treadmill exercise may engage both limb muscles and tongue muscles. Descriptive caption: An outline illustration depicting a rat running, with labels indicating tongue muscles (airway geometry) and limb muscles (running)

Swallowing and breathing both involve coordination of multiple muscles of the upper airway, including the tongue muscles[50]. Exercises that upregulate respiratory activity can therefore activate tongue muscles. For example, treadmill running is an aerobic exercise that activates muscles in the tongue [66], in addition to the expected impacts on limb muscles[73–76] (Fig. 1).

Within the tongue, muscles involved in respiration include the extrinsic muscles of the tongue; the genioglossus (GG) that protrudes and depresses the tongue, and the styloglossus (SG) and hyoglossus (HG) muscles that retract the tongue. A series of studies in anesthetized and tracheotomized rats have shown that extrinsic tongue muscles are activated during inspiration, and co-activation of these muscles may decrease pharyngeal airway collapsibility, thus contribute to shaping airway geometry for respiration [77, 78]. It follows that extrinsic tongue muscles are activated during aerobic exercise to sustain airway patency and maximize airflow. Although notable prior work suggests that the GG muscle may be much less activated by treadmill exercise than other upper airway muscles[79], a prior study demonstrated that treadmill exercise regimen in rats altered contractile properties of GG muscles[66]. Taken together, these findings suggest that treadmill running upregulates activity and induces adaptative changes in hindlimb muscles [73, 80, 81] and extrinsic tongue muscles. Therefore, it is plausible that treadmill exercise paradigms may be used to induce hindlimb and tongue muscle fatigue concurrently in the same experimental animal. This scenario offers opportunities for direct comparisons of measures of muscle fatigue in tongue muscles and hindlimb muscles, thereby potentially revealing possible differential responses to fatigue.

## Biochemical Experimental Measures for the Study of Tongue Muscle Fatigue in Mice and Rats

Aging and exercise paradigms may differentially impact fatiguability of various intrinsic and extrinsic tongue muscles [82]. Biochemical and molecular assays on isolated tongue muscle samples can be used to discern aspects of fatigue, thereby providing a framework for exploring the respective contributions of each muscle to fatigue profiles of the overall muscle system.

Three primary bioenergetic pathways generate energy for muscle contraction (for review, please see [83]):

1. The breakdown of phosphocreatine for immediate generation of ATP,
2. The breakdown of glycogen through glycolysis for the generation of ATP, and
3. Aerobic respiration through mitochondrial oxidative phosphorylation for ATP production.

Muscle fatigue can be experimentally defined as a failure to maintain force or power output as a function of time. Measurement of energy substrate depletion in muscles may reflect an underlying mechanism of fatigue. Muscle ATP content is a direct measure of energy availability. However, ATP turnover in muscle fibers is tightly regulated and subject to multiple homeostatic mechanisms, such that reductions in ATP content during acute fatigue can be small or modest [84, 85]. Glycogen provides an important energy source for ATP production in myofibers, and depletion of glycogen content can also be used as an experimental indicator of muscle fatigue, particularly in the context of sustained exercise [86, 87]. Significant reductions in glycogen content in hindlimb muscles (soleus, plantaris, and vastus lateralis muscles) and diaphragm have been reported immediately following treadmill running [88].

In our laboratory, we have studied the effects of treadmill running on tongue muscle force characteristics and fiber type adaptation[66]. To further study tongue muscle fatigue, we conducted a pilot study of 10 old male F344/Brown Norway rats (31 months of age; 5 treadmill exercise rats and 5 sedentary controls). The treadmill exercise regimen was comprised of one week of acclimation to the stationary treadmill, followed by one week of walking (target speed of 10 cm/s) for 10 min, one week of slow running (target speed of 20 cm/s) for 10 min, and then a final day of faster running to a state of fatigue (30 cm/s). The state of fatigue was behaviorally defined as loss of ability to continue running, at which point rats were euthanized. Food was regulated overnight prior to the final day of treadmill running and euthanasia. Live animal work was approved by the University of Wisconsin Madison Animal Care and Use Committee (IACUC), and in compliance with the Guide for the Care and Use of Laboratory Animals [89].

Muscles were isolated, frozen in liquid nitrogen, and stored at − 80 °C prior to being ground to a fine powder in liquid nitrogen using small pre-cooled mortar and pestle. Aliquots of roughly 10 mg of frozen muscle powder from each sample were frozen at −80 C until subsequent processing for glycogen and gene expression analysis. To evaluate muscle glycogen content, muscle powder samples were homogenized in a volume of 0.03 N HCl normalized to muscle powder weight, then boiled for 5 min, and centrifuged for 10 min to remove insoluble material. Supernatent glycogen content was quantified with a colorimetric plate assay (SIGMA MAK016), as compared to a standard curve. Data were analyzed by unpaired t-tests. For gene expression analysis, RNA was isolated from muscle powder samples through Qiashredder columns and Qiagen Rneasy Fibrous Mini RNA kits. Genomic DNA was removed with Turbo DNAse. A nanodrop spectrophotometer was used to confirm RNA A260/280 values for each sample were between 1.8 and 2.1. Agilent rRNA analysis of a representative SG sample indicated a RIN of 9.1. cDNA was generated using 100 ng RNA from each sample. Muscle gene expression was quantified through qRT-PCR with Taqman primers on a Quantstudio^™^ 7 Flex Real-Time PCR system (Applied Bio-systems). The gene *hprt1* was used as a biological reference gene. Soleus muscle of a rat from non-treadmill conditions that was not otherwise involved in the study provided a biological control reference sample for relative quantification (RQ) calculations. Data were analyzed by unpaired t-tests.

**Fig. 2:**
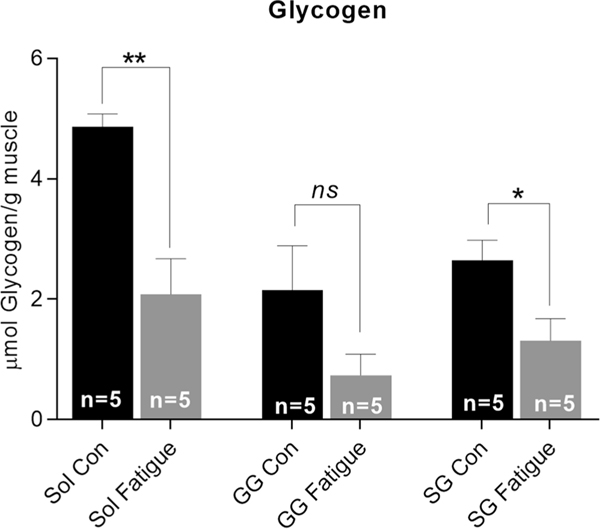
Acute fatigue was shown in both soleus (sol) and styloglossus (SG) muscles through reductions of muscle glycogen in a treadmill exercise condition as compared to control groups (*; *p* < .05), (**; *p* ≤ .01). Tissues were isolated for analysis immediately following exercise. *Con* Control condition, *GG* genioglossus muscle

We found significant reductions in glycogen within the soleus muscle in the hindlimb and within the styloglossus (SG) muscle in extrinsic tongue in rats from the treadmill fatigue conditions, relative to sedentary controls (Fig. 2). The observation of reduced glycogen content in extrinsic tongue muscles immediately following treadmill running complements a prior study that used hypoglossal nerve stimulation to evaluate tongue muscle fatiguability in rats that had completed a treadmill regimen. In that prior study, treadmill running significantly impacted rat tongue muscle fatiguability [66]. Collectively, these points indicate that treadmill running entails increased activation of tongue muscles [66, 90], thereby reducing muscle glycogen content and potentially eliciting acute fatigue in tongue muscles at the time of exercise (Fig. 2), as well as ultimately reducing tongue muscle fatiguability [66].

**Fig. 3:**
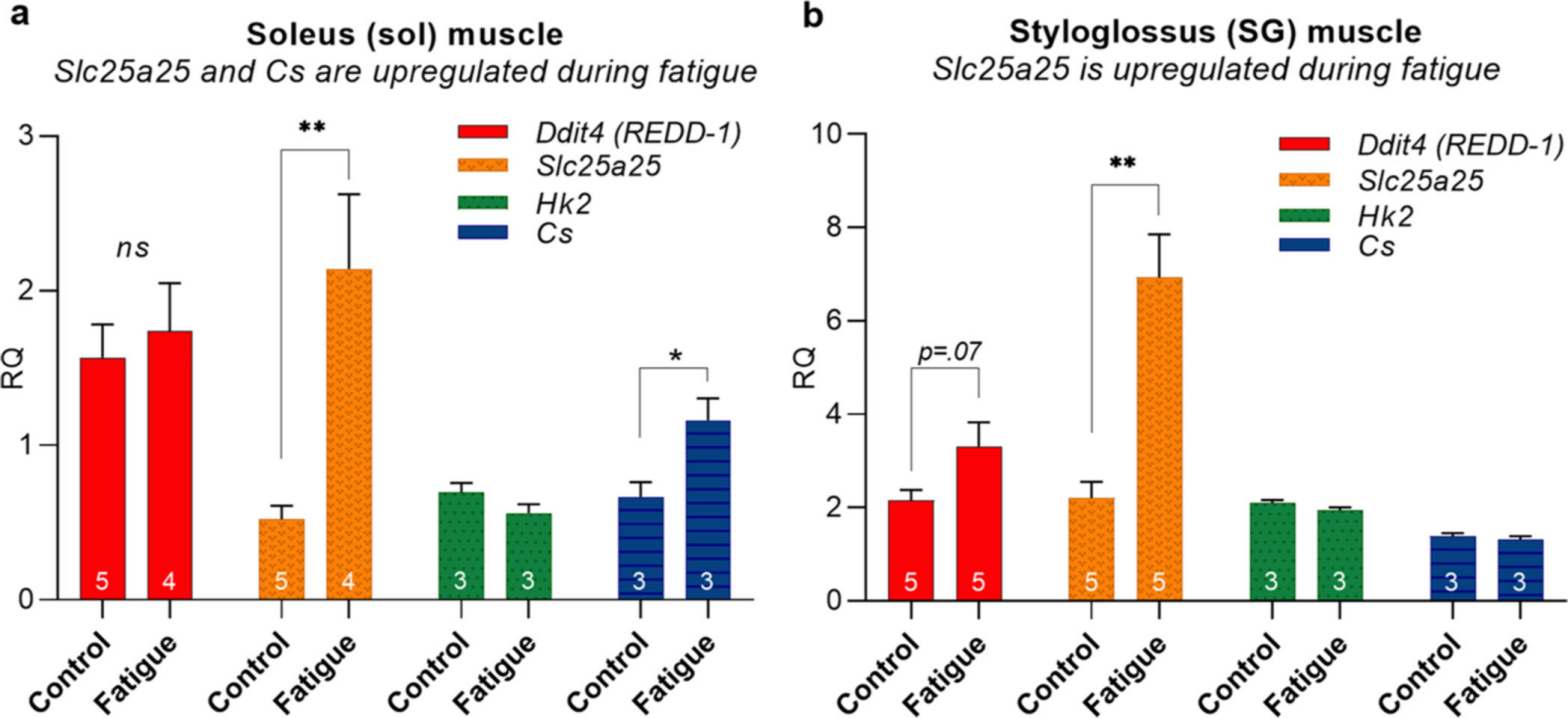
Following treadmill running to a state of fatigue, gene expression of *Slc25a25* in soleus and SG muscles was significantly greater in the fatigue group (**; *p* ≤ .01) than the sedentary control group. Soleus additionally showed higher expression levels of citrate synthase (*Cs*) in the fatigue group than in the control group. (*; *p* < .05). Tissues were isolated for analysis immediately following exercise

Aside from direct measures of energy substrate content in muscles, gene expression profiles in muscle tissue may also be used to obtain an indirect indication of a muscle’s bioenergetic status. Changes in gene expression can happen very quickly and provide a means through which proteins may be upregulated through increased synthesis, or downregulated through decreased synthesis, thereby potentially increasing or decreasing their protein content in the cell. Gene expression profiles may also provide an indication of how a muscle recovers from exercise-induced fatigue, or an indication major bioenergetic pathways are activated [91]. For example, exercise-induced upregulation of genes involved in oxidative capacity and mitochondrial biogenesis expand the capacity of muscle to generate ATP and to thereby meet sustained functional demands [74]. In a small exploratory gene expression study of soleus and SG muscles from the same rats that were evaluated for muscle glycogen content (Fig. 2), qRT-PCR showed significant changes in expression levels of genes relevant to muscle bioenergetic pathways after treadmill running (Fig. 3). This experimentally independent result suggests that the treadmill fatigue regimen induces muscle fatigue in both extrinsic tongue muscle and soleus muscle. In this pilot study, *Ddit4, Slc25a25, Hk2,* and *Cs* (Online Resource Table S1) genes were selected for their relevance to acute fatigue in studies of hindlimb muscle [74, 86, 92, 93].

*Ddit4 (REDD-1)* is a master regulator of gene expression changes that occur after acute aerobic exercise. This was demonstrated in a study of plantaris muscle (a hindlimb muscle) where *Ddit4* mRNA levels increased dramatically after one hour of exercise before returning to baseline levels after 3 h of recovery [93]. *Slc25a25* is an inner mitochondrial membrane solute transporter that is believed to contribute to ATP homeostasis, metabolic efficiency, and physical endurance in treadmill exercise [92]. Hexokinase 2 (*Hk2*) codes for an enzyme responsible for phosphorylating glucose in the first step of glycolysis, and has been shown to upregulate in expression after muscle stimulation to fatigue [86]. Citrate synthase (*Cs*) is an important enzyme in the tricarboxylic (TCA) energy cycle and is widely used as a marker of oxidative capacity of skeletal muscle. *Cs* expression levels have been shown to increase in soleus muscle (a hindlimb muscle) after treadmill exercise [74], thereby likely contributing to an exercise-induced expansion of oxidative bioenergetic capacity of the muscle.

The comparison of hindlimb and tongue muscle fatigue within the same experimental subjects allows examination of differential responses to activity across muscles with distinct functions while controlling for several potentially confounding variables that may influence reproducibility and interpretation of molecular measures of muscle bioenergetics. These variables include timing of sample collection and aspects of sample processing, as well as individual differences across animals. Measures of muscle fatigue and bioenergetic recovery may be exceptionally sensitive to these variables.

### Timing of Sample Collection

Skeletal muscles are exquisitely tuned to maintain bioenergetic homeostasis, and depletion of energy substrates is immediately followed by mechanisms to restore energetic balance [94]. Muscle glycogen content levels illustrate this concept in that glycogen levels can be dramatically reduced during exercise and fatigue, but may quickly rebound [95, 96]. A study of fatigue in tibialis anterior muscle (a hindlimb muscle) demonstrated that restoration of muscle glycogen levels can occur within an hour after fatigue and may rebound to exceed pre-fatigue levels [86] in a phenomena known as supercompensation [86, 95, 96]. The time span of this process can vary widely depending on the nature of the experimental fatigue induction regimen [86]. Gene expression changes responding to muscle fatigue can also occur quickly, with upregulation of some genes occurring immediately during fatigue, after which levels may revert to baseline. Therefore, relative quantities of bioenergetic substrates or markers may vary with time (measured in minutes, hours, or days), as processes of muscle fatigue and recovery unfold. Time frames can vary based on the organism, muscle type, fatigue induction regimen, and detection methods. Interpretation of experiments to evaluate topics related to muscle fatigue may incorporate consideration for this temporal complexity. Some studies of skeletal muscle fatigue and related phenomena associated with exercise are designed with multiple analysis timepoints[86, 93].

Finally, technical aspects of sample collection and sample processing are also important considerations for the study of muscle fatigue. It has been shown that methods of anesthesia and euthanasia in tissue collection from animal models can impact skeletal muscle metabolites [97]. For example, collection of muscle samples following euthanasia as compared to during anesthesia results in significantly different outcomes in measures related to glycolysis [97].

## Knowledge Gaps and Future Work

Avenues for future work may involve development or use of animal models for particular clinical populations to examine mechanisms and treatment of lingual muscle fatigue and dysphagia. An important premise of this research is that it may ultimately lead to the use of optimized exercise strategies to ameliorate tongue muscle fatigue. The use of animal models is beneficial to focus hypotheses for future testing in human subjects in a targeted manner that is based on scientific evidence related to putative mechanisms. The use of human subjects in early studies focused on mechanisms and hypothesis building may not be as fruitful due to variability in access, safety, or other barriers encountered in human subjects research. Murine models offer excellent potential for studying a large array of genetic disorders, developmental differences, aging, and disease processes [98].

Another consideration that should be studied is exercise modality, optimal frequency/dose of the prescribed exercise, and adherence requirements for particular exercises. There may be a great deal of individual variation across clinical populations in the degree to which specific exercise regimens are feasible. Some patients, due to comorbidities or life circumstances, may be disproportionately likely to encounter barriers in accessing traditional progressive resistance tongue exercise. Other patients may be unable to perform aerobic exercises, or may not have access to or be eligible for hypoglossal nerve stimulation. Basic and translational research paradigms that engage with a variety of exercise modalities may be one way to help expand future applicability of research in cranial muscle fatigue and dysphagia to a wider range of demographics and life circumstances.

The biological study of murine tongue muscle fatigue is an interdisciplinary area of interest that integrates perspectives from behavioral, anatomical, physiological, and molecular sciences. The diversity of scientific perspectives involved in the study of tongue fatigue is mirrored by a diversity of potential translational relevance of basic knowledge to be gained from research in this topic. Understanding tongue muscle fatigue is important not only for its implications for management of dysphagia associated with aging, but also because it may be applicable to many other conditions. These may include sleep apnea [62, 99], Down syndrome[100], and Parkinson disease [101]. A better understanding of tongue muscle fatigue may lead to improved management and treatment of a variety of disorders that impact communication and deglutition.

## Supplementary Material

Supplementary Information
