# Supplementary material for: Bioenergetic Evaluation of Muscle Fatigue in Murine Tongue
Source: Dysphagia. 2022 Nov 19;38(4):1039–48. doi: 10.1007/s00455-022-10537-y (PMC10195920; doi:10.1007/s00455-022-10537-y)
Supplement: Supplementary file 1 — Supplementary file1 (DOCX 15 KB) [file 455_2022_10537_MOESM1_ESM.docx]

| **Gene name** | **Rationale** | **Taqman ID#** |
| --- | --- | --- |
| ***DDit4*** (DNA damage inducible transcript 4) | Upregulates in hindlimb muscle after exercise.  A master regulator of exercise-induced metabolic activity in muscle. | *Rn01433735_g1* |
| ***Slc25a25*** | Upregulates in hindlimb muscle after exercise.  Thought to regulate ATP homeostasis in skeletal muscle. | *Rn00595529_m1* |
| ***Hk2*** (Hexokinase 2) | Upregulates in hindlimb muscle after exercise.  Initial enzyme in glycolysis; production of energy from glucose. | *Rn00562457_m1* |
| ***Cs*** (Citrate Synthase) | Upregulates in hindlimb muscle after exercise.  Initial enzyme in the energy-generating tricarboxylic acid (TCA) cycle. | *Rn01774378_m1* |
| ***Hprt1* (**hypoxanthine phosphoribosyltransferase 1) | Used as a reference control (housekeeping) gene. | *Rn01527840_m1* |

**Supplementary Online Resource**

**Table S1. A table showing target genes and corresponding qRT-PCR Taqman Primers**
